# Mental and physical health effects of meaningful work and rewarding family responsibilities

**DOI:** 10.1371/journal.pone.0214916

**Published:** 2019-04-24

**Authors:** Nadya Dich, Rikke Lund, Åse Marie Hansen, Naja Hulvej Rod

**Affiliations:** 1 Department of Public Health, University of Copenhagen. Copenhagen, Denmark; 2 Center for Healthy Aging, University of Copenhagen, Copenhagen, Denmark; 3 Danish National Research Centre for the Working Environment, Copenhagen, Denmark; University o f Genoa, ITALY

## Abstract

Positive feelings about work and family responsibilities benefit psychological well-being, but their physical health effects remain unexplored. The study assessed whether meaningful work and reward from taking care of family benefitted physical health to the same degree as mental health. Participants were 181 Danes aged 49–51. Participants reported on working conditions, providing care to family, depressive symptoms, and perceived stress. Physical health was operationalized as a physiological dysregulation (e.g., hypertension, high levels of blood sugar and cholesterol, high body mass index). A multidimensional index of physiological dysregulation was created using parameters of cardiovascular, metabolic, and immune function. As expected, meaningful work and sense of reward from taking care of family members were associated with better mental health. However, in women, the very same factors were positively associated with higher physiological dysregulation. We conclude that work and family factors promoting psychological well-being may have physical health trade-offs, particularly in women.

## Introduction

Health effects of work and family life have been of interest to researchers for a long time. Numerous studies have linked psychological stress and emotional strain from work and family to adverse health outcomes [1,2]. Recently, however, the interest within psychological theory has shifted towards *positive* health effects of work and family life [3], underscoring the fact that work and family demands not only potentially result in strain, but can also be a source of gratification, meaning, and reward [4–6]. Positive feelings about work and family responsibilities have been shown to benefit mental health and contribute to psychological well-being [5,7,8]. However much less is known about the role of these positive feelings in physical health.

Overall psychological well-being is correlated with better physical health [3,9]. Therefore it is often assumed that the same factors that promote mental health also improve physical health. However, this has not been systematically tested. Studies that do test the effects of work and family on both physical and mental health simultaneously tend to use self-reported physical health. This approach is problematic as subjective ratings of health are to a large degree influenced by the informant’s emotional states, personality, as well as mental health [10]. Furthermore, many physical health problems may start with subtle changes in physiological functioning, which individuals may not always be aware of [11]. Thus, from studies using self-reported health, it is impossible to conclude whether deriving meaning from work and family responsibilities confers the same benefits on objective physical health as it does on mental health.

Moreover, there are reasons to believe that physical health benefits of activities that provide sense of purpose may be less pronounced than their mental health benefits. Indeed, the very nature of such activities suggests a certain amount of effort and even sacrifices of the kind that may take a toll on one’s body. To illustrate, combining work and family responsibilities has been argued to be beneficial for one’s mental health [12]. However, strong commitment to work and family may also sometimes mean that taking care of one’s own physical health is not the highest priority [13,14].

The aim of the present study was to investigate whether positive feelings about work and family responsibilities benefit *objectively measured physical health* to the same extent as they might benefit *mental health*. Specifically, with respect to work, we chose to focus on the perception of work as meaningful. Overall sense of purpose and meaning are important components of psychological well-being. However, with respect to physical health in particular, perception of work as meaningful is one of the least well studied aspects of psychosocial working conditions. Indeed, even though occupational psychology literature is increasingly interested in factors that help employees thrive [15], health literature has been primarily focused on work stress.

With respect to family responsibilities, we focus on the sense of reward from caring for family members. Providing care to children, as well as sick or aged family members, also termed in literature as informal caregiving, is one of the primary family responsibilities. The topic of caregiving has been increasingly gaining attention in the health literature, partly because increasingly more women, who traditionally are the ones to perform the caregiving tasks, are participating in labor market, and partly because the population is ageing, meaning that more elderly people need assistance from their younger relatives. However, as stated above, in the health literature, the focus has been primarily on potential burden and strain associated with caregiving; whether potential psychological benefits from caregiving, such as feeling of reward, translate into physical health benefits has not been extensively examined.

The mental health outcomes of the present study were perceived stress and depressive symptoms. Objectively measured physical health was operationalized as physiological dysregulation, i.e. functioning of physiological systems at a suboptimal level (hypertension is an example of suboptimal functioning of the cardiovascular system). A multidimensional index of dysregulation was created, capturing the functioning of cardiovascular, metabolic, and immune systems. All of these systems have been implicated in the mechanisms linking psychosocial exposures to overt physical disease [16], and individual biological markers of these systems have been studied in relation to psychological work and family factors [17,18]. The advantages operationalizing physical health as a combination of multiple biological parameters are three-fold. They are fully objective as they are based exclusively on laboratory tests. They capture subtle changes in functioning that do not always have overt symptoms and are thus a more comprehensive indicator of physical health problems compared to, e.g. doctor’s diagnosis of a disease. Finally, previous literature has argued for advantages of using a cumulative dysregulation index: even when dysregulation in each individual physiological systems is small and not predictive of health outcomes, the accumulation of adverse physiological changes in the body presents a health risk [19,20].

Previous research has shown that women tend to have more demands related to household and family [21] and are more likely to experience role conflict when combining work and family life [22,23], thus suggesting that the effects of work and family factors on health is likely gender-specific. Therefore in the present study, we also considered gender differences in the association between meaningful work and caregiving reward on one hand and mental and physical health on the other hand.

## Methods

### Participants

The data for the present study were obtained from Copenhagen Ageing and Midlife Biobank (CAMB) [24]. A sub-sample of 203 CAMB participants was recruited during CAMB data collection for an additional study investigating a wide range of stress exposures and their effects on markers of physical health (the “Stress and Health” study). The participants of “Stress and Health” study were all born between 1959 and 1961 at the University Hospital of Copenhagen. The inclusion criteria for the “Stress and Health” study were participation in both CAMB questionnaire and biological tests and no history of myocardial infarction, angina pectoris, stroke, or cancer. The sample size and gender composition were determined a priori as *N* = 200 and 50% women. Furthermore, to ensure a high level of contrast in the exposure to stress, inclusion was also conditioned on the participants’ answer to the question: *“How often have you within the preceding four weeks felt stressed out*?*”* in the CAMB questionnaire with possible answers: *at no time*, *a small part of the time*, *a part of the time*, *a large part of the time*, and *all the time*. The a-priori determined distribution was equal number of individuals with very low (*at no time*) and high (*a large part of the time* or *all the time*) levels of stress in the past four weeks, with 40% in each of these categories and 20% in the remaining medium categories. Once the desired number of participants in each category was reached, data collection for the “Stress and Health” study was stopped. Participants who were not employed were excluded from the present study, leaving the final sample of 94 men and 87 women aged 49 to 51. Written informed consent was obtained from all individual participants included in the study.

### Measures

*Meaningful work* was measured using the 3-item subscale of Copenhagen Psychosocial Questionnaire, a validated instrument to assess psychosocial conditions at work [25]. The items were *Is your work meaningful*?, *Do you feel that the work you do is important*? and *Do you feel motivated and involved in your work*?. Items were rated from 1-*to a very small degree* to 5-*to a very large degree*. The scores for the three items were averaged. Cronbach’s alpha for the meaningful work scale was .76 in the present sample.

Participants were asked if they provided regular care to their parents, spouses, children, grandchildren and other persons. For each of the care recipients, they were asked how many hours a week they spent caring for that person, and how physically and emotionally straining, as well as how rewarding caregiving was. Physical and emotional strain and reward were rated on a 5-point scale from 1-*not at all* to 5-*to a very large degree*. The following variables were created: *average* *caregiving reward*, average *physical strain*, average *emotional strain*, and *total* *caregiving hours* *a week*. We expected that strain and reward from providing care, as well as the time the caregiving responsibilities take, might vary depending on the care recipient (e.g. taking care of children might feel more rewarding than taking care of sick parents, but might also be more time consuming). To account for these differences, the average strain and reward scores were weighted by the number of hours providing care to each recipient. Similar ways to assess caregiving burden have been used in previous literature [26].

*Depressive symptoms* were measured using the Major Depression Inventory, a validated tool for assessing mood [27]. The inventory consists of 12 items describing mood in the past two weeks, e.g., *Have you felt low in spirits or sad*? or *Have you lost interest in your daily activities*?, rated from 0 (*at no time*) to 5 (*all the time*). The items are combined into a single score ranging from 0 to 50.

*Perceived stress* in the past four weeks was assessed using the 10-item version of Cohen’s Perceived Stress Scale. Items, such as *Have you felt nervous and “stressed”*? or “*Have you found that you could not cope with all the things that you had to do*? were rated on a 5-point scale from 1-*never* to 5-very *often*. Items were summed yielding a possible score from 10 to 50.

*Physiological dysregulation* was assessed based on eight biomarkers, reflecting cardiovascular (blood pressure), metabolic (triglycerides, HDL cholesterol, Total Cholesterol, glycated hemoglobin, and BMI), and immune (C-reactive protein, Interleukin-6) activity and have been previously established as clinical predictors of major chronic diseases [11]. Physiological dysregulation score was calculated as a number of markers whose values were beyond clinically established norms, with a possible range from 0 to 8. The exact cut-off values are provided in the S1 Appendix and the laboratory procedure for collecting and analyzing biological samples are described elsewhere [28].

### Statistical analyses

Linear regression was used to assess the effects of work and caregiving responsibilities on depression, perceived stress, and physiological dysregulation. First we assessed the main effects of meaningful work and having any caregiving role in the full sample, controlling for age and SES measured as occupational social class [29]. Further, among those reporting caregiving responsibilities (further referred to as *caregivers*), we also assessed the effects of caregiving reward, as well as all other caregiving-related variables (total caregiving hours, the number of care recipients, and physical and emotional caregiving strain) controlling for age and SES. Finally, in the fully adjusted model, the effect of caregiving reward was adjusted for all other caregiving-related variables in addition to age and SES. The analyses were stratified by gender. Gender differences were also tested as statistical interaction between gender and all of the considered predictors.

## Results

Table 1 shows the distribution of study variables by gender. The majority of participants provided care to at least one person. Sixty-seven percent of caregivers found caregiving rewarding to a *large* or *very large degree*. Only a small fraction of participants reported caregiving to be straining *to a large degree* or *very large degree* (2% for physical strain and 11% for emotional strain). Women and men found their work equally meaningful and 81% of respondents rated their work as meaningful *to a large degree* or *very large degree*. Table 2 shows the proportion of participants taking care of children, parents, spouse, grandchildren and other persons and the strain and reward scores across different groups of care recipients.

**Table 1:** Distribution of study variables by gender.

	Possible Range	Men	Women	*p* for gender difference
**Full sample**		**N = 94**	**N = 87**	
Mean meaningful work score (SD)	1–5	4.2 (0.6)	4.2 (0.6)	0.70
Number (%) of caregivers		58 (62%)	73 (84%)	0.004
**Among caregivers**		**N = 58**	**N = 73**	
Mean nr. of care recipients	1–5	1.7 (0.9)	1.8 (0.8)	.62
Mean nr. of caregiving hrs/wk (SD)		24.6 (30.3)	32.6 (31.6)	0.14
Mean physical strain (SD)	1–5	1.2 (0.5)	1.6 (0.9)	0.002
Mean emotional strain (SD)	1–5	1.6 (0.9)	2.1 (1.3)	0.002
Mean reward (SD)	1–5	3.8 (1.2)	4.0 (1.0)	0.21
Mean depressive symptoms (SD)	1–50	7.8 (7.5)	10.9 (10.0)	0.020
Mean perceived stress (SD)	10–50	20.4 (6.3)	22.4 (7.2)	0.046
Mean physiological dysregulation score (SD)	1–8	1.7 (1.4)	1.2 (1.3)	0.018

**Table 2:** Distribution of family responsibility characteristics by gender and care recipient.

	**Men (N = 58)**				
	Parent	Spouse	Children	Grandchildren	Other
Nr. participants providing care	27 (29%)	26 (28%)	38 (40%)	5 (5%)	18 (19%)
Mean number of caregiving hours/week	2.9 (2.4)	15.4 (13.6)	21.1 (31.2)	2.4 (1.1)	5.6 (9.6)
Mean caregiving physical strain	1.7 (0.7)	1.2 (0.4)	1.2 (0.4)	1.4 (0.9)	1.2 (0.4)
Mean caregiving emotional strain	2.1 (0.9)	1.4 (0.6)	1.6 (0.9)	1.2 (0.4)	1.6 (0.9)
Mean caregiving reward	3.8 (0.9)	3.8 (1.2)	4.3 (0.9)	5.0 (0.0)	3.9 (1.0)
	**Women (N = 73)**				
	Parent	Spouse	Child	Grandchild	Other
Nr. participants providing care	36 (41%)	34 (39%)	53 (61%)	5 (6%)	31 (36%)
Mean number of caregiving hours/week	4.2 (3.6)	18.5 (12.6)	24.5 (27.6)	5.4 (2.7)	7.7 (9.6)
Mean caregiving physical strain	1.7 (0.9)	1.4 (0.8)	1.5 (0.8)	1.2 (0.4)	1.7 (0.9)
Mean caregiving emotional strain	2.4 (1.3)	1.5 (1.0)	2.1 (1.3)	1.2 (0.4)	2.2 (0.9)
Mean caregiving reward	4.0 (0.9)	4.0 (1.1)	4.3 (1.1)	4.8 (0.4)	4.0 (1.2)

Table 3 shows the effects of meaningful work and caregiving responsibilities on depressive symptoms, perceived stress, and physiological dysregulation. For both men and women, higher levels of work meaning were moderately associated with lower levels of depressive symptoms. The direction of the association was the same for perceived stress, but it was not statistically significant. At the same time, in women, but not in men, meaningful work was related to *higher* levels of physiological dysregulation (*p* = .042 for the gender difference). In women, the unstandardized effect of meaningful work was 0.51 (*SE* = 0.24), i.e. the difference between lowest observed meaningful work score (3 = *somewhat* meaningful) and highest observed score (5 = meaningful *to a very large degree*) approximately corresponded to one additional biomarker above a clinical high-risk cut-off.

**Table 3:** Standardized regression coefficients and 95% CIs for the effects of meaningful work and caregiving responsibilities on depressive symptoms, perceived stress and physiological dysregulation.

	Depressive symptoms^a^		Perceived stress^a^		Physiological dysregulation^a^	
Full sample^b^ (94 men, 87 women)	Men	Women	Men	Women	Men	Women
Meaningful work (1 SD increase)	**-0.34 (-0.51; -0.17)**	**-0.31 (-0.53; -0.09)**	**-0.29 (-0.47; -0.11)**	-0.16 (-0.39; 0.07)	-0.05 (-0.23; 0.13)	**0.22 (0.02; 0.42)**
Caregiver (ref = “no”)	-0.01 (-0.35; 0.33)	**0.55 (0.23; 0.87)**	-0.14 (-0.53; 0.25)	0.46 (-0.16; 1.08)	-0.01 (-0.43; 0.41)	0.09 (-0.53; 0.71)
**Among caregivers, adjusted for age and SES only** **(58 men, 73 women)**						
Caregiving reward (1 SD increase)	-0.20 (-0.42; 0.02)	-0.03 (-0.33; 0.27)	**-0.27 (-0.50; -0.04)**	**-0.29 (-0.54; -0.04)**	-0.08 (-0.34; 0.18)	**0.27 (0.03; 0.51)**
Caregiving physical strain (1 SD increase)	0.04 (-0.35; 0.43)	**0.26 (0.01; 0.51)**	-0.22 (-0.57; 0.13)	**0.23 (0.04; 0.42)**	-0.26 (-0.70; 0.18)	-0.09 (-0.30; 0.12)
Caregiving emotional strain 1 SD increase)	-0.05 (-0.38; 0.28)	**0.42 (0.20; 0.64)**	-0.21 (-0.47; 0.05)	**0.32 (0.09; 0.55)**	0.18 (-0.18; 0.54)	-0.19 (-0.39; 0.01)
Nr. of caregiving hours/wk (10 hrs increase)	-0.03 (-0.11; 0.05)	-0.02 (-0.11; 0.07)	-0.03 (-0.08; 0.02)	-0.01 (-0.09; 0.07)	0.02 (-0.07; 0.11)	0.04 (-0.03; 0.11)
Nr. of care recipients (1 unit increase)	-0.06 (-0.33; 0.21)	-0.16 (-0.49; 0.17)	-0.12 (-0.35; 0.11)	-0.22 (-0.48; 0.04)	0.14 (-0.17; 0.45)	**0.32 (0.05; 0.59)**
**Among caregivers, adjusted for age and SES; all caregiving characteristics are mutually adjusted for** **(58 men, 73 women)**						
Caregiving reward (1 SD increase)	-0.23 (-0.49; 0.03)	-0.02 (-0.25; 0.21)	**-0.26 (-0.51; -0.01)**	**-0.29 (-0.55; -0.03)**	-0.06 (-0.30; 0.18)	**0.25 (0.06; 0.44)**
Caregiving physical strain (1 SD increase)	0.29 (-0.08; 0.66)	0.00 (-0.31; 0.31)	0.08 (-0.44; 0.60)	0.01 (-0.26; 0.28)	**-0.65 (-1.06; -0.24)**	0.06 (-0.14; 0.26)
Caregiving emotional strain 1 SD increase)	-0.17 (-0.48; 0.14)	**0.46 (0.06; 0.86)**	-0.23 (-0.65; 0.19)	**0.31 (0.03; 0.59)**	**0.56 (0.19; 0.93)**	-0.14 (-0.35; 0.07)
Nr. of caregiving hours/wk (10 hrs increase)	-0.01 (-0.08; 0.06)	0.01 (-0.08; 0.10)	0.00 (-0.09; 0.09)	0.03 (-0.05; 0.11)	0.02 (-0.08; 0.12)	-0.01 (-0.10; 0.08)
Nr. of care recipients (1 unit increase)	-0.04 (-0.26; 0.18)	0.11 (-0.22; 0.44)	-0.08 (-0.36; 0.20)	-0.03 (-0.37; 0.31)	0.22 (-0.07; 0.51)	0.23 (-0.10; 0.56)

^a^Standardized effects are presented to enable comparison of the effect sizes between outcomes and between predictors.

^b^Adjusted for age and SES

Reporting any caregiving responsibilities was strongly related to higher depressive symptoms in women, but not in men (*p* = .003 for the gender difference). The effect was approximately the same in size for perceived stress in women, but was not statistically significant. Neither in men, nor in women reporting caregiving responsibilities per se was associated with physiological dysregulation.

Among caregivers, reward was moderately associated with lower perceived stress in both men and women. At the same time, in women, but not in men, reward was also associated with *higher* levels of physiological dysregulation (*p* = 0.055 for gender differences). These results held regardless of whether other aspects of caregiving were controlled for.

Several other factors related to providing care predicted mental health and physiological dysregulation. In the fully adjusted model, emotional strain was related to higher depressive symptoms and perceived stress in women (*p* = 0.007 and *p* = 0.008 for gender differences for depressive symptoms and perceived stress respectively). Physiological dysregulation was positively related to emotional strain and negatively related to physical strain in men, but not in women (*p* = 0.19 and *p* = 0.12 for gender differences for physical and emotional strain respectively). Controlling for other aspects of caregiving, the time participants spent providing care to others and the number of care recipients was not related to any of the outcomes, either in men, or in women.

## Discussion

Deriving sense of purpose from one’s professional and family life has long been recognized as an important component of thriving and well-being [3,30]. Studies have confirmed that the pursuit of meaning and engagement contributes to happiness and enhances life quality, life satisfaction, and mental health [31,32]. Because psychological well-being and physical health are not independent from one another [3,9], it is often assumed that the same factors that promote psychological well-being and mental health should also contribute to better physical health. However, our findings suggest that this may not always be the case. The results of the study show that, especially in women, *the very same* factors that appear to be protective of mental health, namely meaningful work and caregiving reward, are associated with higher levels of physiological dysregulation.

The exact reasons why factors that contribute to sense of meaning and reward would take a toll on the body remain to be investigated. It seems plausible, however, that that the effects of meaning of work and reward from family responsibilities may be non-linear: both low and high levels might have a negative effect on health, but for different reasons. The low levels might act as a psychological stressor as any effort going into work and family would not be compensated by satisfaction (*cf*. effort-reward imbalance model, according to which the mismatch between effort spent at work and rewards received may elicit a stress response) [33]. The negative effects of psychological stress on the body have been well-documented [34,35]. At the same time, very high levels of meaning and reward might be a sign of over-commitment. With respect to work, researchers have speculated that very high levels of engagement might interfere with recovery, jeopardizing health [36,37]. Furthermore, a curvilinear relationship between time spent for paid work and psychological distress and time spent with a spouse and psychological distress has been reported [38]. However, because most participants in our sample rated their work as meaningful or highly meaningful and caregiving as rewarding or highly rewarding, we would have not been able to detect this non-linearity. Thus, the negative effects of meaning and reward on physiological functioning may only be telling part of the story.

Our findings also need to be considered in the context of timing of the exposure. Physiological dysregulation resulting from psychosocial exposures takes time to develop and is usually a result of long-term circumstances [16]. Therefore, if the findings of our study reflect a causal effect of work and family factors on physiological dysregulation, this effect is likely due to a long-term exposure rather than the concurrent life situation. Alternatively, our findings might be a reflection of certain personality traits, such as a tendency to overcommit, which would result in both high levels of engagement with work and family, but also, over time, in physiological dysfunctioning.

The above caveats notwithstanding, our findings of adverse effect of caregiving reward on physiological functioning among women are in line with recently published evidence that parental empathy, while associated with greater self-esteem and purpose in life for parents, also has its physiological costs in terms of increased levels of low-grade inflammation [39]. Taken together, these findings suggest that pursuit of meaning and purpose by engaging with work and family, which makes life worth living [3], may physically wear people out and compromise health. Importantly, however, we only observed negative health effects of meaningful work and caregiving reward in women. Gender differences in health effects of employment have long been known, and the health benefits of being gainfully employed alongside family responsibilities tend to be more pronounced among men, while women are more likely to experience role conflict and role strain from combining work and family duties [22,23].

Our study has a number of strengths and limitations. We had access to detailed measures of various aspects of caregiving responsibilities and their subjective perception, a measure of meaningful work, one of the psychosocial aspects of work that is least researched in health literature, as well as a wide range of biomarkers. The availability of biomarker data has allowed us to overcome one of the major limitations of previous literature and investigate the effect of work and family life on *both* mental health and *objective* physical health as the outcome.

At the same time, because of the high costs associated with collecting detailed psychosocial as well as biological information, the sample was relatively modest in size. We were able to detect moderate to strong associations in our data, but may have not had enough power to detect associations more modest in size and interactions between work and family factors. Furthermore, the sample was highly selected. As described in the CAMB cohort profile, CAMB participants had slightly higher education than the general population [40]. Furthermore, in the “Stress and Health” study, participants with high levels of stress were over-sampled by design, which means that this sample is not representative of the general population in terms of stress levels. At the same time, most of the participants did not experience high emotional or physical strain from caregiving, low caregiving reward or low work meaning. Thus we were not able to investigate the whole range of these exposures. Moreover, participants represented a very narrow age range, 49 to 51. The content of both family roles and of work changes with age. Therefore our findings may not generalize to younger adults.

Perception of meaning of work, strain and reward from family, as well depressive symptoms and perceived stress, are inherently subjective and can only be measured by self-reports. Due to that, and because of the cross-sectional design of the study, the causality of these associations cannot be established with certainty, i.e. both perception of meaning may affect mental health symptoms, as well as mental health can affect how individuals perceive their work and family life. Furthermore, both the perception of one’s work and family life and mental health may be influenced by a wide range of confounding factors, such as current emotional state, recent stressful experiences, and personality traits. From that perspective, both good mental health and the ability to see meaning and reward in one’s daily activities may just be different facets of overall psychological well-being.

Furthermore, selection bias may partly explain the observed relationships between work and family factors on one hand and the objectively measured physiological functioning on the other hand. For instance, it is possible that women who are aware of their sub-optimal physical health are more appreciative of their work and family roles, thus explaining the association between work meaning and caregiving reward on one hand and physiological dysregulation on the other hand. Exclusion of participants with pre-existing major chronic disorders reduces the likelihood of selection bias; however, it cannot be completely ruled out. Importantly, however, given that our results were very different for mental vs. physical health outcomes, it seems highly unlikely that either unmeasured confounding or selection bias are the only explanations for the observed associations.

Despite the outlined limitations, the findings of the study underscore the necessity of a broad and comprehensive definition of health and for multi-methodological assessment of health outcomes in research investigating the role of psychosocial work and family factors in health. On the one hand, self-reported measures of health are prone to bias and may not provide the full picture of individuals’ well-being. On the other hand, the sole reliance on objectively measured physiological parameters does not provide a full picture either. Many health researchers and practitioners argue that subjective satisfaction with one’s life and one’s health and good psychological adjustment to whatever health conditions one might have is as important as objective indicators of health [41]. If our divergent results for mental and physical health are replicated in larger samples and longitudinal designs, we believe that both mental and physical health consequences of engagement in work and family need to be taken into account in clinical practice.

Female employment has been a politically charged topic and extra care should be taken when interpreting potentially controversial results presented herein. We believe the findings suggest that women participating in labor market and invested in their careers might benefit most from policies aimed at improving work-life balance and from interventions helping workers prioritize down-time and physical well-being.

## Supporting information

S1 Appendix Clinical cut-off points used to compute the physiological dysregulation index and the proportion of participants beyond the clinical cut-off point. (DOCX)
