# Supplementary material for: Mental and physical health effects of meaningful work and rewarding family responsibilities
Source: PLoS One. 2019 Apr 24;14(4):e0214916. doi: 10.1371/journal.pone.0214916 (PMC6481914; doi:10.1371/journal.pone.0214916)
Supplement: S1 Appendix — (DOCX) [file pone.0214916.s001.docx]

APPENDIX. Clinical cut-off points used to compute the physiological dysregulation index and the proportion of participants beyond the clinical cut-off point.

| Biological marker | Clinical cut-off | Proportion of participants beyond the clinical cut-off point |
| --- | --- | --- |
| Systolic blood pressure /  Diastolic blood pressure | 140 / 90 mmHg | 16% |
| C-reactive protein | 3 mg/L | 23% |
| Ttriglycerides | 2.26 mmol/L | 20% |
| HDL cholesterol* | 1.03 mmol/L | 7% |
| Total cholesterol | 6.2 mmol/L | 50% |
| Glycated hemoglobin (HbA1c) | 6.5% | 1% |
| Body Mass Index | 30 | 17% |
| Inetrleukin-6** | 2.92 pg/ml | 15% |

* Low values of this biomarker (i.e. below the clinical cut-off) represent risk.

** Because no established clinical cut-off point exists for IL6, we used the 75^th^ percentile as a cut-off (based on the entire CAMB sample).
